# HEMOPHAGOCYTOSIS BY BLASTS IN A CHILD WITH ACUTE MONOCYTIC LEUKEMIA AFTER CHEMOTHERAPY

**DOI:** 10.1590/1984-0462/2021/39/2019290

**Published:** 2020-07-03

**Authors:** Mariela Granero Farias, Priscila Aparecida Correa Freitas, Fabiane Spagnol, Meriene Viquetti de Souza, Ana Paula Alegretti, Mariluce Riegel, Adriano Nori Rodrigues Taniguchi, Liane Esteves Daudt

**Affiliations:** a Hospital de Clínicas de Porto Alegre, Porto Alegre, RS, Brazil.; b Universidade Federal do Rio Grande do Sul, Porto Alegre, RS, Brazil.

**Keywords:** Acute monocytic leukemia, Hemophagocytic lymphohistiocytosis, Hemophagocytic syndrome, Macrophage activation syndrome, Leucemia monocítica aguda, Linfo-histiocitose hemofagocítica, Síndrome hemofagocítica, Síndrome de ativação macrofágica

## Abstract

**Objective:**

To describe the case of a child who presented hemophagocytic lymphohistiocytosis (HLH) associated with acute monocytic leukemia after chemotherapy, with hemophagocytosis caused by leukemic cells.

**Case description:**

In a university hospital in Southern Brazil, a 3-year-old female was diagnosed with acute monocytic leukemia with normal karyotype. The chemotherapy regimen was initiated, and she achieved complete remission six months later, relapsing after four months with a complex karyotype involving chromosomes 8p and 16q. The bone marrow showed vacuolated blasts with a monocytic aspect and evidence of hemophagocytosis. The child presented progressive clinical deterioration and died two months after the relapse.

**Comments:**

HLH is a rare and aggressive inflammatory condition characterized by cytopenias, hepatosplenomegaly, fever, and hemophagocytosis in the bone marrow, lymph nodes, spleen, and liver. Although rare, malignancy-associated HLH (M-HLH) is fatal. The patient in this case report met five out of the eight established criteria for HLH. The evolution of the patient’s karyotype, regardless of the diagnostic profile, seemed secondary to the treatment for acute monocytic leukemia. In this case, the cytogenetic instability might have influenced the abnormal behavior of leukemic cells. This is a rare case of HLH in a child with acute monocytic leukemia.

## RESUMO

**Objetivo:**

Descrever um caso de um paciente pediátrico que apresentou linfo-histiocitose hemofagocítica (LHH) associada à leucemia monocítica aguda pós-quimioterapia, com hemofagocitose causada pelas próprias células leucêmicas.

**Descrição do caso:**

Em um hospital universitário do Sul do Brasil, uma menina de três anos foi diagnosticada com leucemia monocítica aguda com cariótipo normal. Após receber protocolo quimioterápico, atingiu remissão seis meses depois do início do tratamento, recaíndo quatro meses após com um cariótipo complexo envolvendo ambos os cromossomos, 8p e 16q. A medula óssea mostrava-se infiltrada por células blásticas vacuolizadas com aspecto monocítico, com evidências de hemofagocitose. A criança apresentou um declínio clínico progressivo e dois meses após a recaída foi a óbito.

**Comentários:**

A LHH é uma condição inflamatória rara e agressiva caracterizada por citopenias, hepatoesplenomegalia, febre e hemofagocitose na medula óssea, linfonodos, baço e fígado. A LHH associada a doenças malignas, embora seja uma condição rara, é potencialmente fatal. A paciente deste caso apresentou cinco dos oito critérios estabelecidos para o diagnóstico de LHH. A evolução do cariótipo do paciente, independentemente do perfil do diagnóstico, pareceu ser secundária ao tratamento da leucemia monocítica aguda, sendo que a instabilidade citogenética pode ter influenciado o comportamento atípico observado nas células leucêmicas. Este é um dos raros casos de LHH em uma criança com leucemia monocítica aguda.

## INTRODUCTION

Acute myeloid leukemia (AML) accounts for about 20% of the childhood leukemia cases.^1^ Over the last decades, the survival rate of children with AML has significantly improved, and estimates indicate that around 60% of them have been cured in most developed countries.^2^ ^,^ ^3^ In children aged 0-2 years, AML has been associated with a high prevalence of unfavorable prognosis and increased risk of treatment-related toxicity, with acute monocytic leukemia (AMoL) being one of the most common AML subtypes in infants.^3^ Despite the advances in the treatment of children with leukemia, AMoL continues to be responsible for high rates of morbidity and mortality.^2^

AML diagnosis requires morphological, immunophenotypic, and molecular evaluation, as well as the presence of certain cytogenetic abnormalities related to age, incidence of unbalanced aberrations, and complex karyotypes.^4^ AML with t(8;16)(p11;p13) is an example of such abnormalities, defined by a unique gene expression signature, monocytic morphology, high frequency of leukemia cutis, and erythrophagocytosis in childhood.^5^ ^,^ ^6^

Reports of hemophagocytic lymphohistiocytosis (HLH) in cases of childhood AML (especially AMoL) are very rare in the literature, corresponding mainly to hemophagocytosis caused directly by leukemic cells. HLH is a rare and aggressive inflammatory condition characterized by cytopenias, hepatosplenomegaly, fever, and hemophagocytosis in the bone marrow (BM), lymph nodes, spleen, and liver. HLH is diagnosed by a combination of at least five of the following eight criteria: fever, splenomegaly, cytopenia, hypertriglyceridemia and/or hypofibrinogenemia, hemophagocytosis, low or absent NK cell activity, hyperferritinemia, and increased levels of soluble CD25.^7^ This disorder results from two distinct reasons: (1) Primary or familial HLH that occurs during the first years of life, being fatal when not treated;^7^ ^,^ ^8^ ^,^ ^9^ or (2) Secondary or reactive HLH associated with underlying immunological or malignant diseases.^8^ ^,^ ^10^ ^,^ ^11^

The pathogenesis of HLH was recently defined as the impaired activation of T lymphocytes following the stimulation by immune responses, which results in large amounts of inflammatory cytokines that promote macrophage infiltration and cytokine network formation.^12^

Malignancy-associated HLH (M-HLH) may occur concomitantly with a neoplasm or during chemotherapy, mainly in patients who are already in remission.^13^ ^,^ ^14^ Children and infants present M-HLH more often in lymphomas and solid neoplasms.^10^ ^,^ ^14^

In this paper, we described a case of HLH with blast phagocytosis in a child with relapsed AMoL after chemotherapy.

## CASE REPORT

In 2014, a previously healthy 3-year-old white Brazilian female patient was admitted to the Hospital de Clínicas de Porto Alegre with fever and abdominal pain. Imaging examination showed discrete amounts of pleural effusion on the left and right lungs and enlarged spleen. Laboratory results indicated pancytopenia (leukocyte count: 1.37×10^9^/L, lymphocytes count: 1.17×10^9^/L, hemoglobin: 42 g/L, and platelet count: 25×10^9^/L), high levels of C-reactive protein (69.6 mg/dL), and lactate dehydrogenase (2,280 U/L). Screening tests for hepatitis B surface antigen (HBsAg), toxoplasma IgG/IgM, and Venereal Disease Research Laboratory (VDRL) were negative. BM aspirate slide review showed blast infiltration with a monoblastic aspect. Immunophenotypic analysis identified two populations with abnormal phenotype: (1) 28% of immature cells positive for CD64, CD4, HLADR, CD117, CD56 bright, myeloperoxidase dim, CD11b, CD65, CD15 bright, CD38 bright, and CD45 dim, and negative for CD34, CD14, CD36, CD13, and NG2; and (2) 46% of more differentiated cells presenting a similar immunophenotype, but positive for CD14 and negative for CD117. Karyotype analysis showed 46XX[20] chromosomes and absence of FMS-like tyrosine kinase 3 (FLT3) mutation. Therefore, the patient was diagnosed with AMoL without chromosomal abnormality.

The treatment, in accordance with the 2004 Berlin-Frankfurt-Munster (BFM) chemotherapy protocol,^15^{Creutzig, 2013, Development of a curative treatment within the AML-BFM studies} was initiated as follows: first AIE induction (cytarabine/idarubicin/etoposide); second HAM induction [high-dose of cytarabine (3 g/m^2^)/mitoxantrone] after forty-two days; and AI consolidation [cytarabine (0.5 g/m^2^)/idarubicin] three months after the start of treatment. The patient presented clinical worsening one month after febrile neutropenia and received cefepime. A second HAM cycle (1 g/m^2^/mitoxantrone) was administered five months after the first induction. A new episode of febrile neutropenia occurred after central catheter placement, and cefepime and vancomycin therapy was restarted, with no signs of invasive fungal infection on radiographic examinations or galactomannans. Central culture was positive for coagulase-negative *staphylococci*. The child was in complete remission one month after the second HAM. Next, intensification HAE [high dose of cytarabine (3 g/m^2^)/etoposide] was initiated, and the maintenance cycle (mercaptopurine/cytarabine) started after forty-five days, along with radiotherapy 12Gy.

**Figure 1:**
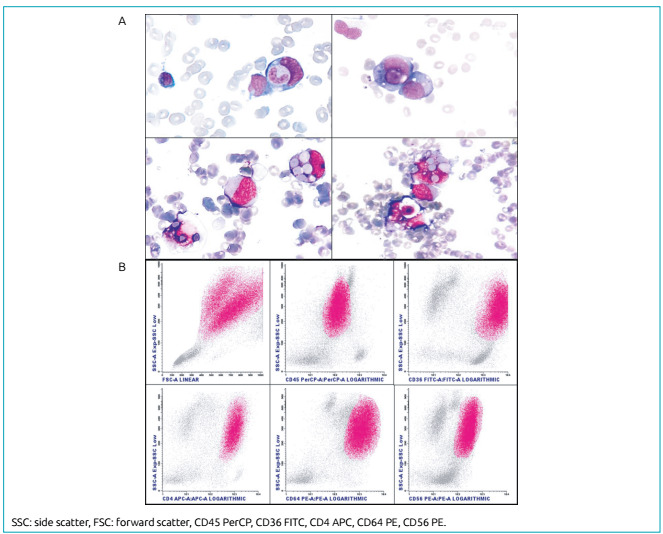
Bone marrow film showing acute monocytic leukemia and phagocytosis in leukocytes and erythrocytes (A). Immunophenotypic profile of the leukemic population. Immature cells show high side scatter (SSC) due to phagocytic activity and cytoplasmic vacuoles (B).

**Figure 2:**
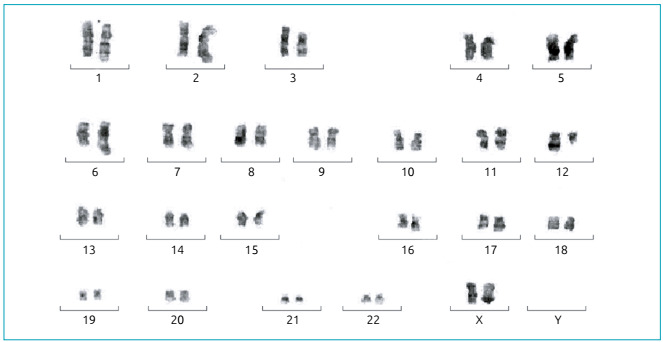
GTG-banded karyotype of the proband showing complex rearrangements partly involving chromosomes 1p, 2p, 3p, 6q, 8p, 10qter, 16q, and 17q.

Four months after remission, the patient presented hematemesis, petechiae, splenomegaly, and fever; she also showed pancytopenia (leukocyte count: 2.68×10^9^/L, blast count: 0.56×10^9^/L, hemoglobin: 73 g/L, and platelet count: 37×10^9^/L); high levels of lactate dehydrogenase (5,640 U/L), ferritin (107.6 nmol/L), and C-reactive protein (31.3 mg/dL); and normal levels of triglycerides (100 mg/dL) and fibrinogen (214 mg/dL). Screening tests for HbsAg, VDRL, toxoplasma, cytomegalovirus, and Epstein-Barr virus IgM were negative, whereas toxoplasma, cytomegalovirus, and Epstein-Barr virus IgG showed positive results. A new BM aspirate slide review revealed 81% of vacuolated blasts along with hemophagocytosis. Immunophenotypic analysis indicated 66% of neoplastic cells presenting very high side scatter (SSC), which may be a result of the vacuoles and phagocytic activity in these cells. Neoplastic cell populations expressed CD64 bright, CD36 bright, CD4, HLADR dim, CD56, myeloperoxidase, CD11b, CD13 dim, CD65, CD15 bright, and CD45 dim, but they did not express CD34, CD117, CD14, CD16, or NG2 (Figure 1). The cytogenetic study revealed a complex karyotype involving nine chromosomes into 13 cells (Figure 2). Unfortunately, cells in suspension were not available to confirm the complex chromosome rearrangement by fluorescence *in situ* hybridization (FISH) analysis.

Subsequently, the patient started a chemotherapy regimen with high-dose cytarabine/fludarabine/idarubicin/etoposide/filgrastim. Cefuroxime, vancomycin, and cefepime were prescribed to treat fever, neutropenia, palpitations, and shortness of breath. Blood culture, in that period, identified a catheter-related growth of coagulase-negative *Staphylococcus*. One week later, a new blood culture was negative for microorganism growth. However, the patient had a fever of unknown origin, and, immediately, meropenem and fluconazole were introduced, with the maintenance of vancomycin for ten days. Nevertheless, the patient presented progressive clinical deterioration. Blasts increased to 98% in the BM after four weeks. Dexamethasone therapy (6 mg/m^2^/day) was started due to persistent hemophagocytosis. A new blood culture showed growth of multidrug-resistant *Klebsiella pneumoniae*, and the child received several antibiotics (polymyxin B, gentamicin, vancomycin, and amphotericin). The patient died two months after the onset of worsening.

## DISCUSSION

The association between malignancies and HLH may be related to direct immune activation by transformed lymphocytes and/or loss of inhibitory immune function. Many genes involved in HLH are also associated with an increased risk of several neoplasms. Therefore, M-HLH should not preclude a complete genetic evaluation.^16^

The largest multicenter study in children with M-HLH was performed in Turkey and reported its association with acute lymphoblastic leukemia (66.6% of cases), AML (7.4% of cases), Hodgkin lymphoma (HL), non-HL, rhabdomyosarcoma, neuroblastoma, and Langerhans cell histiocytosis. This study showed that HLH occurred predominantly during leukemia treatment.^10^ A cohort study of 21 children revealed that mature T-cell disease was the most frequent M-HLH. It also identified that only two children with HLH had AML (one presenting AML with maturation and the other was not specified) during chemotherapy and after remission.^14^ Data from an Austrian study, including 508 children with several types of malignancies, showed that six children developed HLH during antineoplastic treatment, and two of them had AML with maturation.^8^ Another study conducted in Austria reported that children with AML developed HLH significantly more often than patients with acute lymphoblastic leukemia.^17^

The description of AML cases associated with HLH in children is uncommon in the literature and seems to be even rarer for AMoL. Lackner et al. have suggested a predisposition of this subtype of malignancy towards the development of HLH, once they found a 30% prevalence of HLH in children with AMoL, against a 4.6% prevalence in other AML cases. In their study, three children with AMoL who developed HLH felt the first symptoms after the first BFM 2004 protocol cycle.^2^

HLH during chemotherapy frequently occurs in patients who have already achieved remission and could be a result of the immune suppression caused by the treatment, which might trigger fatal infections.^14^ Moreover, in some cases of leukemia associated with HLH, blasts may perform phagocytosis directly, instead of the mature phagocytic cells.^15^ The pathogenic mechanism related to this behavior in neoplastic cells remains unclear, although associations have been found with some chromosomal abnormalities such as t(16;21) and t(8;16).^18^ ^,^ ^19^ ^,^ ^20^

This behavior was present in blasts of the patient presented in this case report and could be attributed to the complex cytogenetic aberrations acquired after treatment, including chromosomes 8p and 16q.

The karyotype evolution, irrespective of diagnosis, seems secondary to the AML treatment.^20^ Even normal karyotypes can become highly unstable and turn into complex karyotypes during the progression of the disease.^21^

Regarding the immunophenotypic expression of leukemic cells, it was positive for CD56 antigen - a cell adhesion molecule present in NK/T lymphoma, multiple myeloma, and some subtypes of AML.^18^ ^;^ ^22^ There was overexpression of CD56 associated with AMoL in the diagnosis, which maintained positivity after disease recurrence, although with lower intensity. A meta-analysis by Xu et al. reported this antigen overexpression as an adverse prognostic factor in AML.^22^ Aside from the extramedullary involvement, CD56 may influence survival and remission duration, and has also been related to HLH and vacuolation in AML cases presenting t(16;21).^18^

Decreased NK activity and high levels of soluble interleukin 2 receptor (sCD25) are useful markers for HLH diagnosis and are typically present in infants and children.^22^ These tests were not performed due to unavailability in our laboratory routine. Given the evaluation of the available results and following the current diagnostic guidelines, the patient met the respective criteria for HLH. The phagocytic activity of blasts in the BM, along with the development of karyotype abnormalities and infections secondary to chemotherapy, might have led to the poor prognosis of our patient.

In this study, we described a case of HLH caused directly by AMoL blasts with complex cytogenetic aberrations after the patient underwent chemotherapy. In conclusion, HLH in pediatric patients with malignant neoplasms remains a challenge due to its importance and diagnostic difficulty, reflected in the high mortality rates.
